# Therapeutic Effect of Alpha-Pinene on In Vitro and In Vivo Models of Mild Traumatic Brain Injury

**DOI:** 10.3390/life16071110

**Published:** 2026-07-02

**Authors:** Harry Jung, Tae Yeon Kim, Dong Hyuk Youn, Sung Woo Han, Jong-Tae Kim, Youngmi Kim, Chulho Kim, Jong-Hee Sohn, Jae-Jun Lee, Jong-Kook Rhim, Jin Pyeong Jeon

**Affiliations:** 1 Institute of New Frontier Research, Hallym University College of Medicine, Chuncheon 24252, Republic of Korea; harry_88@naver.com (H.J.); solar3833@gmail.com (T.Y.K.);; 2 Department of Neurology, Hallym University College of Medicine, Chuncheon 24253, Republic of Korea; 3 Department of Anesthesiology and Pain Medicine, Hallym University College of Medicine, Chuncheon 24253, Republic of Korea; 4 Department of Neurosurgery, Jeju National University College of Medicine, Jeju 63241, Republic of Korea; 5 Department of Neurosurgery, Hallym University College of Medicine, Chuncheon 24253, Republic of Korea; 6 Department of Population and Quantitative Health Sciences, Umass Chan Medical School, Worcester, MA 01655, USA

**Keywords:** traumatic brain injury, alpha-pinene, cognitive impairment, neuroinflammation

## Abstract

We aimed to investigate the therapeutic effects of alpha-pinene (α-pinene), a major component of phytoncide, in in vitro and in vivo models of mild traumatic brain injury (mTBI), raising the possibility of treating mTBI, a condition currently lacking adequate therapies. An in vitro model was established using SH-SY5Y cells and a cell injury controller, and was treated with α-pinene (0.5 g/mL). An in vivo model was induced by a stereotaxic impactor in male C57BL/6J mice and treated with α-pinene intravenously (50 mg/kg and 100 mg/kg) for 3 days post-injury. Histopathological and immunohistochemical comparisons were conducted alongside cognitive function tests to evaluate -pinene treatment. In vitro analysis showed that alpha-pinene treatment significantly increased TUNEL-positive cells. Elevated NOX4 and p22phox mRNA expressions and a high Bax/Bcl-2 protein expression ratio were noted following alpha-pinene treatment. mTBI mice treated with alpha-pinene exhibited a notable decrease in brain water content with fewer FJB-positive neurons and lower protein expression of Bax and Bcl-2 compared to untreated mTBI mice. Immunofluorescence staining for NOX4 and GFAP-positive or Iba-1-positive cells demonstrated that the increased oxidative stress and astrogliosis or activated microglia triggered by mTBI were alleviated after alpha-pinene treatment. Cognitive function testing revealed a general improvement in mTBI mice treated with alpha-pinene, with statistical significance observed in the NOR test. Alpha-pinene appears to be beneficial for neuroprotection and enhancing cognitive function in the early phases of mTBI.

## 1. Introduction

Alpha-pinene (α-pinene) is a bicyclic monoterpene and the main component of phytoncide. Traditionally, α-pinene has been used to treat various inflammatory conditions such as bronchial inflammation, gout, and diarrhea in Northeast Asia [1]. In addition, α-pinene has been used in steam inhalation therapy for respiratory symptoms associated with bronchial inflammation, colds, and coughs, supporting its potential translational applicability in humans [2]. Additionally, it has served as a terpene-based medicine for relieving neuropathic pain, insomnia, and mood disorders [3]. In neurological disorders, α-pinene has demonstrated beneficial effects on cerebral ischemia and cognitive impairment. Tumor necrosis factor-α (TNF-α) and interleukin-1ß (IL-1ß) levels were reduced in the cortex and hippocampus in a cerebral ischemia–reperfusion injury model following α-pinene treatment [4]. Lee et al. [5] found that α-pinene significantly improved scopolamine-induced cognitive deficits, accompanied by an increase in choline acetyltransferase activity. Despite the potential therapeutic effects of α-pinene, it has not yet been adopted clinically as an alternative for antiplatelet agents or cognitive enhancers, primarily due to the absence of large-scale, data-driven prospective studies to determine if α-pinene outperforms existing medications. Moreover, given the severity of the conditions it may treat, there remains a hesitancy to employ α-pinene in clinical practice, indicating a need to identify specific, less severe conditions where current treatments are inadequate or unsatisfactory.

Mild traumatic brain injury (mTBI), defined as a condition in which the Glasgow Coma Scale (GCS) score is between 13 and 15, with loss of consciousness lasting less than 30 min, and post-traumatic amnesia not exceeding 24 h or a transient neurological deficit [6,7] is the most prevalent type of TBI. Coupled with a surge in physical activities among the young and an increasing elderly population, incidences of mTBI have risen from 569.4 in 2006 to 807.9 in 2012 per 100,000 population in the United States [8]. Despite the classification as mild, mTBI can present a broad spectrum of symptoms, ranging from those that dissipate within months to persistent neurological dysfunctions or even mortality [6,9]. In particular, individuals with comorbidities, those who experience hypotension upon admission, or those above 65 often exhibit worsening conditions similar to moderate-to-severe TBI [10]. However, a major challenge in the current management of mTBI is the lack of effective treatments for post-injury headache and cognitive impairments. Current strategies often involve merely prescribing non-steroidal anti-inflammatory drugs (NSAIDs) [11], despite their neuroprotective properties, signaling a significant gap in drug development for these symptoms in clinical practice. Excitotoxicity from TBI increases intracellular Ca^2+^ and reactive oxygen species (ROS) levels. Notably, NADPH oxidase (NOX) serves as a principal ROS source, exacerbating neuronal apoptosis and inflammation in the brain [12]. Consequently, targeting ROS reduction by inhibiting NOX may represent a valuable approach for addressing TBI-related conditions inadequately managed by traditional treatments [13]. Ma et al. [12] showed that NOX4 deletion significantly reduced lesion size and neural cell death while diminishing oxidative stress. In cerebral microvascular endothelial cells (CMVEC), knockdown of NOX4 using small interfering RNA revealed protection against oxidative stress and apoptosis triggered by TNF-alpha, though this was not observed with NOX-2 [14]. Based on these findings, our research focuses on the potential therapeutic benefits of α-pinene in neuroprotection and cognitive repair by targeting NOX4 activity in in vitro and in vivo models of mTBI.

## 2. Materials and Methods

### 2.1. Study Design and Experimental Groups

For the in vitro experiments, SH-SY5Y cells were divided into four groups: normal, normal + α-pinene (0.5 μg/mL), mTBI (injury), and mTBI + α-pinene (0.5 μg/mL). For the in vivo experiments, male C57BL/6J mice were randomly assigned to normal and mTBI groups, with 15 mice in each group. The mTBI group was further subdivided into the following treatment groups: normal, mTBI + DMSO, mTBI + α-pinene (50 mg/kg), and mTBI + α-pinene (100 mg/kg). All treatments were administered intravenously. Randomization was performed using a predefined allocation sequence, and investigators responsible for outcome assessments were blinded to group assignments.

### 2.2. Reagents and Drug Preparation

(-)-α-pinene (purity 99%) was purchased from Sigma-Aldrich (St. Louis, MO, USA). A stock solution was prepared by diluting (-)-α-pinene in dimethyl sulfoxide (DMSO; Sigma-Aldrich, St. Louis, MO, USA). Prior to use, the stock solution was further diluted with culture medium for in vitro experiments or sterile saline for in vivo administration to obtain the desired final concentrations. For in vivo experiments, the final injection volume was adjusted to 100 μL per mouse. The final concentration of DMSO was maintained at 0.1% (*v*/*v*) in all experimental groups, including vehicle controls.

### 2.3. Cell Culture and In Vitro mTBI Model

An in vitro model of mTBI was established using the human neuroblastoma cell line SH-SY5Y, as previously described [15,16]. SH-SY5Y cells were maintained in a culture medium composed of equal volumes of MEM and Ham’s F-12, supplemented with fetal bovine serum (10%) and penicillin (100 U/mL). Cultures were maintained in a humidified incubator supplied with 5% CO_2_ at 37 °C. Cells were plated onto BioFlex 6-well culture plates (Flexcell International Corporation, McKeesport, PA, USA) and grown to 80–90% confluence. Mechanical injury was induced using a Cell Injury Controller II system (Flexcell International Corporation, McKeesport, PA, USA) with a pulse duration of 50 ms and a pressure setting of 4.0. Immediately after injury, α-pinene was added at a concentration of 0.5 μg/mL, and cells were incubated for 24 h before analysis. This concentration was chosen based on cell viability tests conducted with 0.25 and 0.5 μg/mL, guided by earlier studies [4].

### 2.4. Cell Viability Assay

To assess cell viability, SH-SY5Y cells were distributed into 96-well plates at 2.5 × 10^5^ cells/cm^2^ and analyzed using the cell counting kit-8 (CCK-8). After 24 h of α-pinene treatment, CCK-8 solution was added to each well and allowed to react for 30 min. Optical density was subsequently determined at 450 nm using a GloMax Explorer Multimode reader (Promega, Madison, WI, USA). Each assay was independently repeated at least three times, with three replicate wells per condition. Non-adherent cells were removed and the remaining cells were exposed to α-pinene at two different concentrations (0.25 and 0.5 μg/mL). The viability reagent (CCK-8; Dojindo Molecular Technologies, Inc., Rockville, MD, USA) was subsequently applied to each well and allowed to react for 30 min before measurement. Absorbance was measured at a wavelength of 450 nm using a microplate reader (GloMax Explorer Multimode, Promega, Madison, WI, USA). Each experiment was conducted independently at least three times with triplicate samples.

### 2.5. Quantitative Real-Time PCR

Quantitative real-time PCR (qRT-PCR) was performed to assess mRNA expression. Total RNA was extracted from cells using easy-BLUE solution (Invitrogen, Carlsbad, CA, USA). cDNA was synthesized from total RNA using random primers and the Maxime RT PreMix kit (INtRON Biotechnology, Seongnam, Republic of Korea) according to the manufacturer’s instructions. Gene expression analysis was performed by qRT-PCR using the primers shown in Table S1. Amplification was carried out with an initial denaturation at 94 °C for 5 min, followed by 45 cycles of denaturation at 94 °C for 30 s, annealing at 55 °C for 30 s, and extension at 70 °C for 15 s [16]. Relative levels were determined using the 2^−ΔΔCt^ method after normalization of GAPDH.

### 2.6. In Vivo mTBI Model and Drug Administration

An mTBI model was generated for male C57BL/6J mice, aged seven to eight weeks, using a stereotaxic impactor (RWD Life Science, Shenzhen, China) during anesthesia (2.5% isoflurane). The specific targeting site was M/L = −2.0 mm and A/P = −1.5 mm at a depth of 1.5 mm. A 2 mm blunt tip was employed for modeling at a velocity of 3.0 m/s and a dwell time of 0.5 ms (Figure 2A,B). These impact parameters are consistent with previously established mild TBI models, which induce limited tissue damage and low mortality compared to moderate TBI [16,17,18]. All experimental procedures received approval from the Institutional Animal Care and Use Committee (IACUC) of Hallym University (approval no. Hallym2021–54). Mice were housed under standard laboratory conditions (22 ± 2 °C, 50–60% humidity, 12 h light/dark cycle) with *ad libitum* access to food and water. Animals were monitored daily for general health and signs of distress throughout the experimental period. Human endpoints were established according to the IACUC guidelines. No animals reached the humane endpoints during the study. The experimental mice were randomly assigned to four groups: normal, mTBI + DMSO, mTBI + α-pinene (50 mg/kg), and mTBI + α-pinene (100 mg/kg), with 30 mice in each group. The normal group was used as a baseline control. α-pinene was administered intravenously via the tail at 15 min after injury induction and then once daily for three consecutive days (days 1–3), for a total of four injections. At the end of the experimental period, mice were euthanized under deep anesthesia using isoflurane overdose, and brain tissues were collected for subsequent analyses. Animals were sacrificed on day 3 after injury.

### 2.7. Western Blot Analysis

Western blotting was performed to evaluate protein expression levels in both SH-SY5Y cells and brain tissue samples. Total proteins were extracted using RIPA buffer supplemented with protease and phosphatase inhibitors (100:1 ratio, GeneDEPOT, Katy, TX, USA), and protein concentration was determined prior to analysis. Equal amounts of protein (20 μg) were mixed with 5 × SDS sample buffer (ELPIS-Biotech, Daejeon, Republic of Korea) and boiled at 99 °C for 10 min. Samples were separated on 10–15% polyacrylamide gels and transferred onto membranes at 100 V for 50–60 min. Membranes were blocked with 5% bovine serum albumin (BSA) in 1 × TBST buffer for 1 h at room temperature. Primary antibodies against NOX4 (Abclonal, Woburn, MA, USA), Bax (Cell signaling Technology, Danvers, MA, USA), Bcl-2 (Cell signaling Technology, Danvers, MA, USA), and β-actin (Santa Cruz Biotechnology, Dallas, TX, USA) were diluted 1:1000 in 5% BSA buffer and applied to the membranes overnight at 4 °C. After washing three times with 1 × TBST buffer for 5 min each, membranes were incubated with horseradish peroxidase (HRP)-conjugated goat anti-rabbit IgG and goat anti-mouse IgG (Enzo Life Sciences, Farmingdale, NY, USA) at a dilution of 1:2500 for 1 h at room temperature. Chemiluminescent images were acquired following incubation with an HRP detection reagent (Thermo Fisher Scientific, Waltham, MA, USA) using an Amersham^TM^ ImageQuant^TM^ 500 (Cytiva, Incheon, Republic of Korea). Band intensities were quantified using ImageJ 1.49v software (National Institutes of Health, Bethesda, MD, USA), and target protein levels were normalized to β-actin.

### 2.8. Fluoro-Jade B Staining

Brain tissue slides were hydrated sequentially in 100% ethanol for 3 min, 70% ethanol for 1 min, and distilled water for 1 min, and were then incubated for 15 min in 0.06% potassium permanganate at room temperature. Subsequently, the slides were stained under light-blocking conditions using 0.001% Fluoro-Jade B (FJB) solution (Histo-Chem Inc., Jefferson, AR, USA) for 30 min. Following staining, the slides were rinsed three times for 1 min in distilled water and air-dried at room temperature for at least 10 min. The slides were then mounted using dibutyl phthalate polystyrene xylene (Sigma-Aldrich Co., Louis, MO, USA). Stained brain tissues were observed using a fluorescence microscope within a wavelength range of 450–490 nm (Carl Zeiss, Oberkochen, Germany) [19].

### 2.9. Immunofluorescence Staining

Cryo-sectioned brain tissues were hydrated in distilled water for 1 min and immersed three times in 1 × PBS for 5 min at room temperature. Endogenous peroxidase activity was quenched with 3% H_2_O_2_ in 100% methanol, followed by antigen retrieval in boiling 1 × TE buffer (10 mM Tris-HCl, 1 mM EDTA, pH 8.0). Tissues were then blocked using 5% horse serum for 60 min and incubated overnight at 4 °C with primary antibodies against NOX4 (1:250, CUSABIO TECHNOLOGY LLC, Houston, TX, USA), GFAP (1:250, Abcam, Cambridge, UK), and Iba-1 (1:200, Wako Chemicals USA, Richmond, VA, USA). The tissues were subsequently incubated with secondary antibodies, including goat anti-rabbit Alexa Fluor 488 (Invitrogen, Carlsbad, CA, USA) and donkey anti-rabbit Alexa Fluor 594 (Invitrogen, Carlsbad, CA, USA). Fluorescence microscopy was performed using wavelength ranges of 450–490 nm and 594–590 nm (Carl Zeiss, Oberkochen, Germany) [19].

### 2.10. Behavioral Assessment

Cognitive function was assessed using the novel object recognition (NOR), Y-maze, and the Morris water maze (MWM) tests, as previously described [19,20]. Detailed procedures for each behavioral test are provided in the supplementary methods.

### 2.11. Statistical Analysis

Experimental results are expressed as mean values with their corresponding SEM. Statistical comparisons among groups were performed using one-way analysis of variance (ANOVA) followed by Bonferroni’s post hoc test [21]. Statistical significance was defined as * *p* < 0.05, ** *p* < 0.01, *** *p* < 0.005, and **** *p* < 0.001 [16,22]. All analyses were conducted using GraphPad Prism version 8.0 (GraphPad Software Inc., San Diego, CA, USA).

## 3. Results

### 3.1. Cytoprotective Effects of α-Pinene in the In Vitro mTBI Model

**Figure 1:**
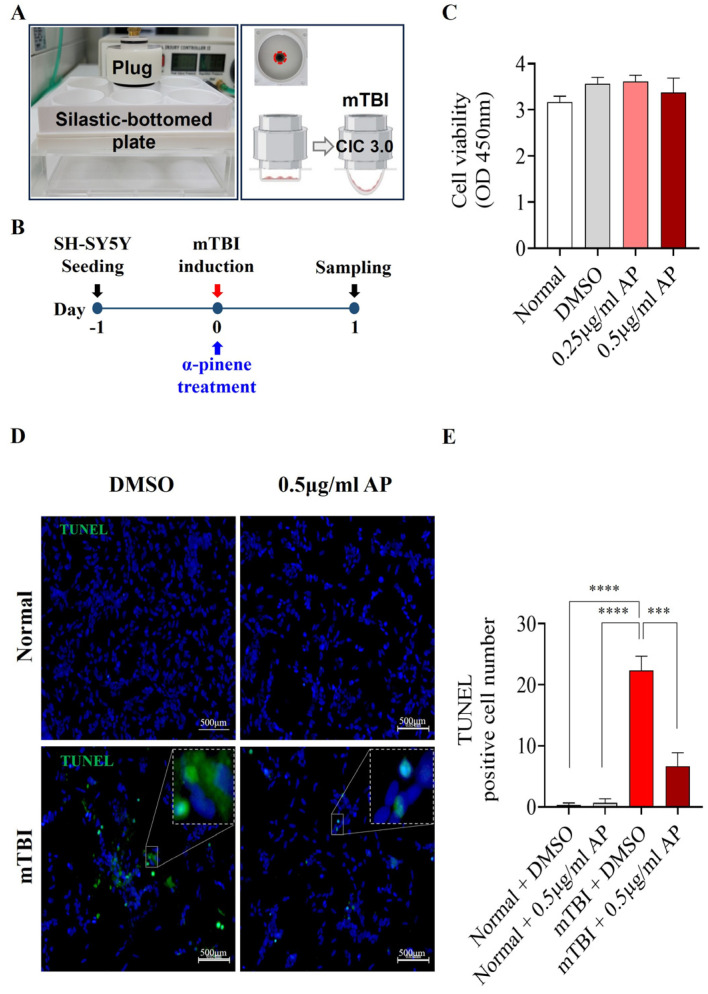
Experimental design and cell viability analysis in the in vitro mTBI model. (**A**,**B**) An in vitro model using a cell injury controller system and the experimental design. (**C**–**E**) Cell viability assays and TUNEL staining to assess neuronal cell death. Scale bar: 500 μm, *** *p* < 0.005 and **** *p* < 0.001. Data represent mean ± standard error of the mean (SEM).

CCK-8 assay indicated no significant decrease in cell viability across the different treatment groups, confirming that the concentrations of α-pinene used were not cytotoxic to SH-SY5Y cells (Figure 1C). Analysis of TUNEL-positive cells demonstrated a significant reduction in positive cells following treatment with α-pinene (0.5 μg/mL) (Figure 1D,E).

### 3.2. Modulation of Oxidative Stress and Apoptosis by α-Pinene

**Figure 2:**
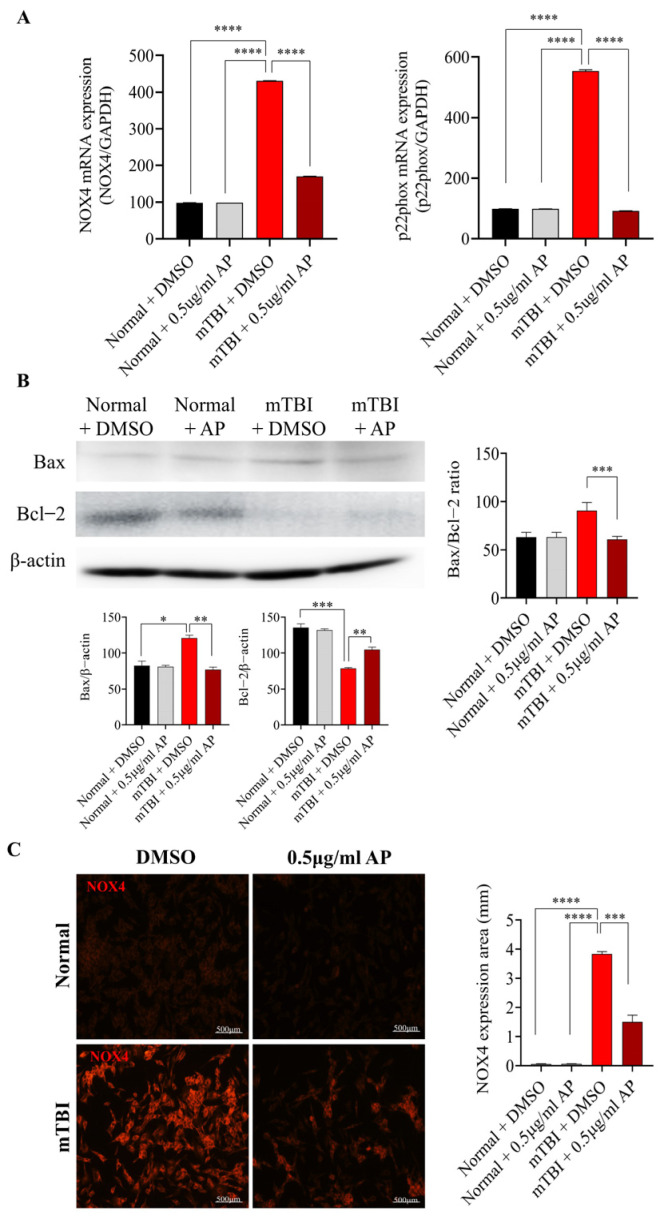
Alterations in oxidative stress and apoptosis-related markers following α-pinene treatment in the in vitro mTBI model. (**A**) mRNA expressions of NOX4 and p22phox. (**B**) Representative Western blot images and quantitative analyses of Bax and Bcl-2 protein expression normalized to β -actin as well as protein expression of the Bax/Bcl-2 ratio. (**C**) Representative immunofluorescence image and quantitative analyses of NOX4 expression in neuronal cells following α-pinene treatment. Scale bar: 500 μm, *** *p* < 0.005 and **** *p* < 0.001. Data represent mean ± standard error of the mean (SEM).

α-pinene treatment mitigated the increased levels of mRNAs of NOX4 and p22phox observed after trauma, highlighting α-pinene’s role as a ROS-mitigating agent (Figure 2A). Following mTBI, Bax expression increased whereas Bcl-2 expression decreased, resulting in a higher Bax/Bcl-2 ratio. α-pinene treatment significantly reduced Bax expression, restored Bcl-2 expression, and lowered the Bax/Bcl-2 ratio (Figure 2B). Moreover, immunohistochemical analysis revealed a marked decrease in the expression area of NOX4 after treatment with α-pinene (Figure 2C).

### 3.3. Neuroprotective Effects of α-Pinene on an In Vivo Model of mTBI

**Figure 3:**
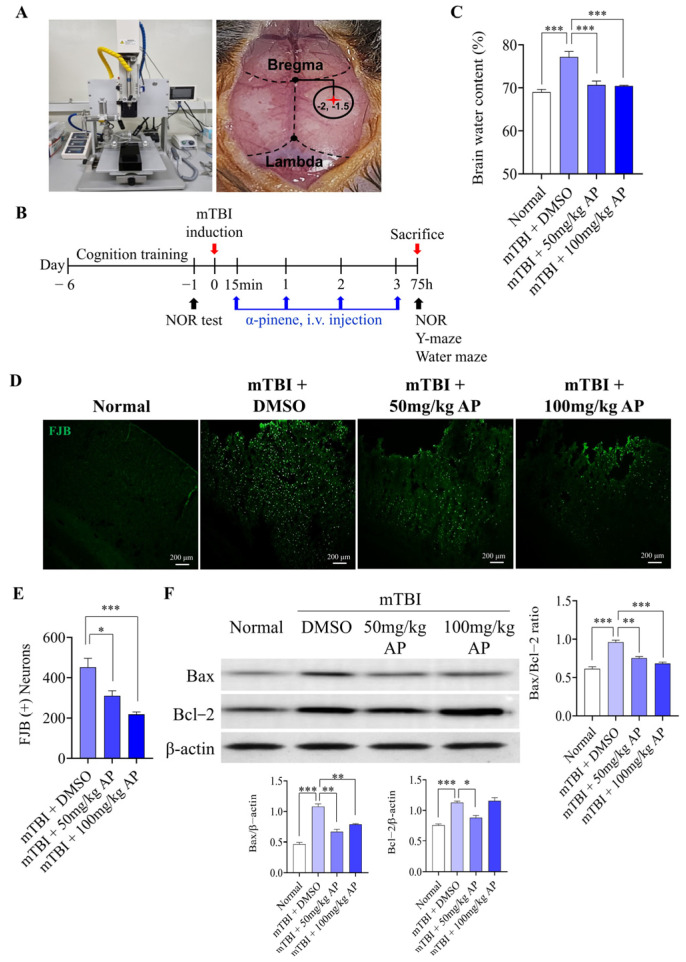
Histological evaluation following α-pinene treatment in the in vivo mTBI model. (**A**,**B**) An in vivo model using controlled cortical impact and the described experimental design. (**C**) Evaluation of brain water content analysis. (**D**,**E**) Representative FJB staining and quantification of FJB-positive neurons. (**F**) Representative Western blot images and quantitative analyses of Bax and Bcl-2 protein expression normalized to β-actin, together with the Bax/Bcl-2 ratio in brain tissues. Scale bar: 200 μm * *p* < 0.05, ** *p* < 0.01 and *** *p* < 0.005. Data represent mean ± standard error of the mean (SEM).

Following mTBI induction, histopathological analyses and cognitive tests were conducted. Compared to the normal control group, mTBI led to increased brain water content; however, α-pinene treatment at doses of 50 or 100 mg/kg resulted in lower brain water content than in mice treated with DMSO (Figure 3C), indicating α-pinene’s therapeutic potential in reducing cerebral edema post-injury. Although α-pinene treatment reduced the number of FJB-positive neurons, no significant dosage-dependent difference was observed (Figure 3D,E). The levels of apoptosis-related proteins Bax and Bcl-2 were significantly altered following mTBI, indicating activation of apoptotic signaling. Compared with the normal control group, both Bax and Bcl-2 expression levels were increased after injury, resulting in an elevated Bax/Bcl-2 ratio. Treatment with α-pinene reduced Bax expression and attenuated the increase in the Bax/Bcl-2 ratio. Notably, Bcl-2 expression showed a differential response to α-pinene treatment, being lower in the 50mg/kg group but higher in the 100mg/kg group compared with the DMSO-treated mTBI group (Figure 3F).

### 3.4. Inhibition of Neuroinflammation and NOX4 Signaling

**Figure 4:**
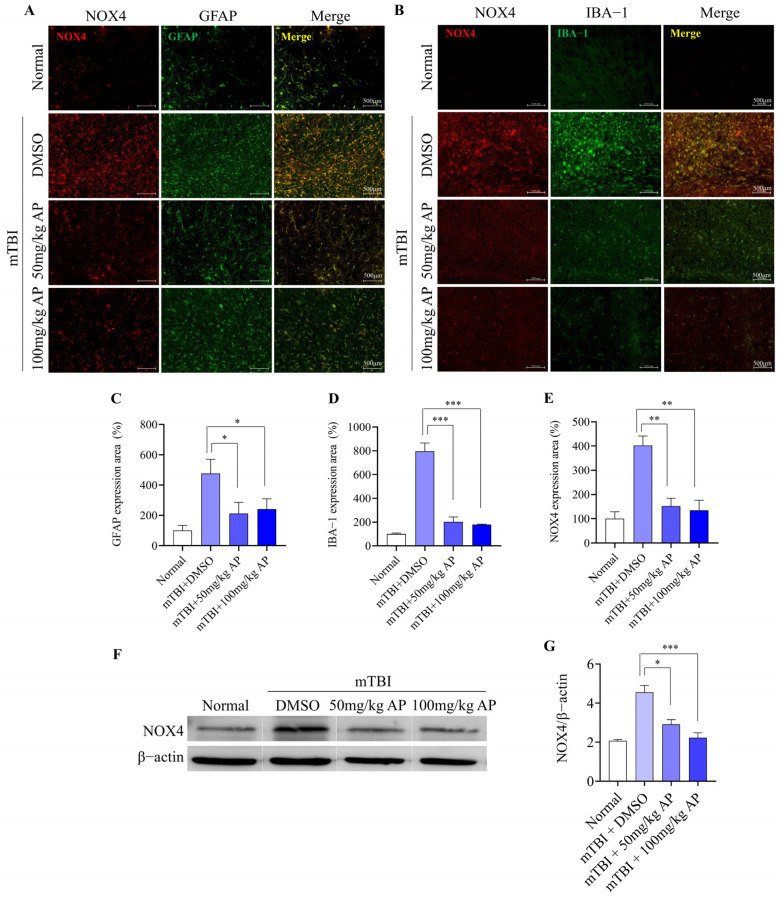
Glia activation and NOX4 expression following α-pinene treatment in the in vivo mTBI model. (**A**–**E**) Representative immunofluorescence staining and quantitative analysis of NOX4 expression in GFAP- or Iba-1-positive cells. (**F**,**G**) Representative Western blot analysis of NOX4 in the brain tissues and its quantification. Non-adjacent lanes from the same original membrane were spliced for presentation. Splice sites are indicated by thin dividing lines. * *p* < 0.05, ** *p* < 0.01, and *** *p* < 0.005. Data represent mean ± standard error of the mean (SEM).

Immunofluorescent staining for NOX4 (red) and GFAP-positive or Iba-1-positive cells (green) showed that mTBI induced an upregulation in NOX4 alongside GFAP or Iba-1, indicating increased oxidative stress and the presence of either astrogliosis or activated microglia (Figure 4A,B). Compared to the normal control group, increased expressions of GFAP, Iba-1, and NOX4 were observed in TBI with DMSO. Treatment with α-pinene at 50 or 100 mg/kg decreased these expressions (Figure 4C–E). Western blot analysis also showed that NOX4 expression in the damaged brain was alleviated after α-pinene treatment, although the reduction was not significantly different between the dosages of 50 and 100 mg/kg (Figure 4F,G).

### 3.5. Improvement of Cognitive Function Following α-Pinene Treatment

**Figure 5:**
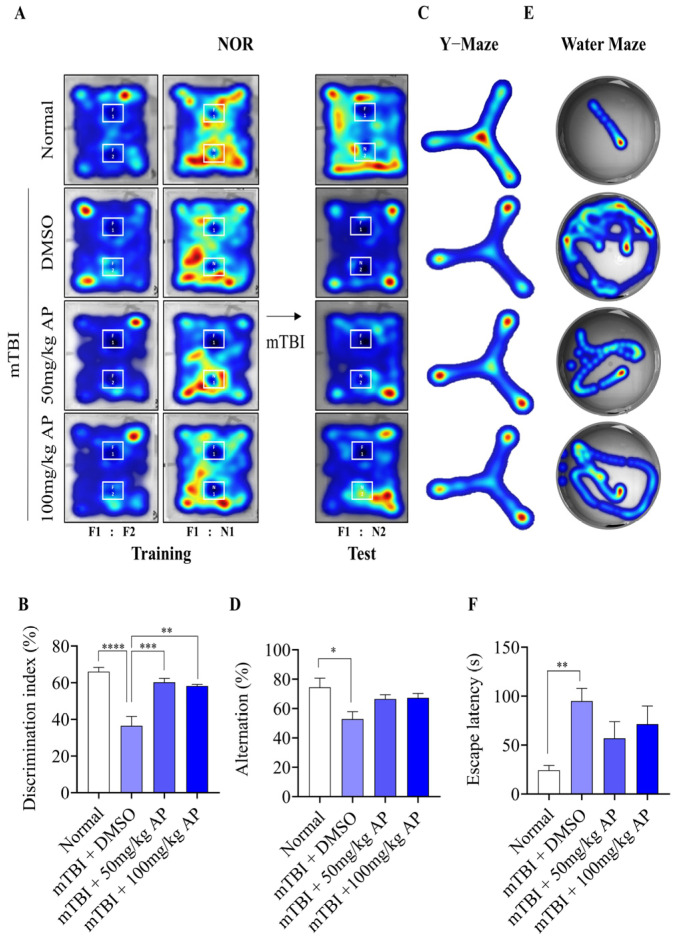
Behavioral assessment following α-pinene treatment in the in vivo mTBI model. Cognitive function was evaluated using the NOR (**A**,**B**), Y-maze (**C**,**D**), and MWM tests (**E**,**F**). Heatmaps represent the spatial distribution of animal activity, with red indicating high activity, yellow/green indicating intermediate activity, and blue indicating low activity. * *p* < 0.05, ** *p* < 0.01, *** *p* < 0.005, and **** *p* < 0.001. Data represent mean ± standard error of the mean (SEM).

Overall, mTBI resulted in an increased discrimination index in NOR and elevated escape latency in MWM tests, alongside a decreased alteration rate in the Y-maze test. Specifically, the change in the discrimination index observed in the NOR test was most pronounced following brain injury. Treatment with α-pinene at doses of 50 or 100 mg/kg in TBI mice significantly restored the discrimination index, indicating that α-pinene effectively mitigates cognitive impairment during the acute phase of mTBI (Figure 5A,B). The Y-maze test assessing spatial working memory showed that TBI mice treated with α-pinene exhibited improved alteration rates compared to those treated with DMSO, though the difference was not statistically significant (Figure 5C,D). The MWM test results paralleled those of the Y-maze, with α-pinene treatment leading to reduced escape latency, suggesting enhanced spatial learning and memory post-injury, though these improvements were not statistically significant when compared to untreated TBI mice (Figure 5E,F).

## 4. Discussion

**Figure 6:**
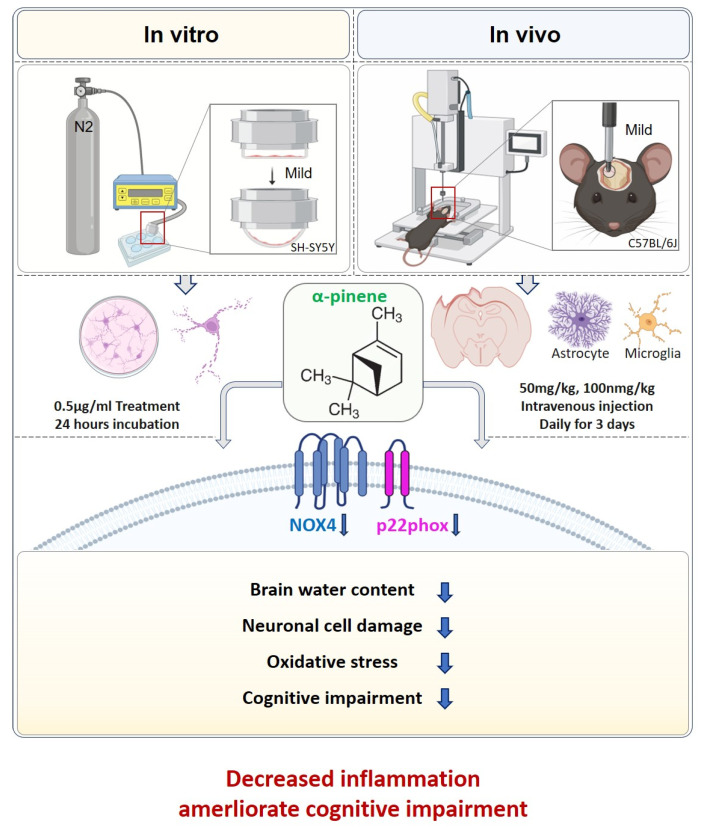
Schematic overview of the experimental workflow and proposed mechanism of α-pinene in mTBI.

Our study suggested that α-pinene treatment immediately after injury can aid in recovering from cognitive impairment during the acute phase by mitigating NOX4-linked ROS damage and neuroinflammation following mild severity of TBI, which is often encountered in clinical practice but insufficiently addressed and treated. In both in vitro and in vivo models of mTBI treated with α-pinene, there was an observed improvement in brain water content, neuronal cell damage, oxidative stress, and cognitive impairment, coupled with a notable reduction in NOX4 expression (Figure 6).

The significance of TBI, particularly of mild severity, tends to be underestimated in clinical settings due to perceptions of it as a minor condition with few major abnormalities on imaging tests and a lower likelihood of requiring surgical intervention compared to moderate or severe TBI. Patients with mTBI are generally advised to rest and may be prescribed painkillers to manage acute pain. However, as many as 20% of patients with mTBI experience persistent pain and cognitive dysfunction [23]. Tham et al. [24] reported that 57% of adolescents with mTBI experienced pain greater than 3 points on a numerical rating scale, even 36 months post-injury. Additionally, early cognitive impairment within the first 2 weeks post-injury commonly occurs, resulting in discomfort for the patients [25]. Such ongoing pain and cognitive impairment are significant factors that diminish the quality of life following mTBI. Despite this, the prevailing treatment approach—rest and administration of anti-inflammatory drugs—remains inadequate, highlighting the need for alternative treatment strategies.

Interest in forest therapy, also known as forest medicine, has surged due to its demonstrated benefits in enhancing immunity and improving physical and mental health [26]. α-pinene is the most commonly encountered terpene in nature and serves as the main constituent of phytoncide. α-pinene has demonstrated protective effects such as antioxidation, anti-apoptotic, and anti-neuroinflammatory effects in acute brain injuries or chronic neurodegenerative diseases [4,27]. Khoshnazar et al. [4] observed reduced levels of neuroinflammation in the hippocampus, cortex, and striatum, alongside reduced blood–brain barrier permeability following α-pinene treatment. Further, α-pinene treatment resulted in a decrease in neuronal loss in CA1 and altered expressions of brain-derived neurotrophic factor (BDNF), tropomyosin-like receptor kinase B (TrkB), and cAMP response element binding protein (CREB) in the hippocampus in a rat model of kainic acid-induced memory impairment [27]. Based on these findings, we proposed that α- pinene could serve as an alternative approach for treating patients suffering from mTBI, particularly during the acute phase post-injury. mTBI may not cause notable structural damage directly from trauma, but subsequent injuries can induce cellular dysfunction and initiate chemical changes, leading to ROS production and neuroinflammation [13]. Since NOX enzymes are primary contributors to ROS generation, selectively inhibiting NOX represents a promising strategy for addressing TBI-related neurological impairments [28]. Of the NOX subtypes, NOX4 is notably widespread and highly expressed in the central nervous system [28,29]. Therefore, our investigation into the activity of NOX4 pre and post α-pinene treatment indicated that immediate post-injury α-pinene administration may help reduce neuronal apoptosis, neuroinflammation, and cerebral edema by inhibiting NOX4-related pathways. However, ROS production was not directly measured in the present study, and further studies using ROS-specific assays are required to confirm the relationship between α-pinene, NOX4 regulation, and oxidative stress. In terms of cognitive deficits, patients with mTBI receiving α-pinene treatment showed improvements in recognition tasks, unlike those observed in the Y-maze and MWM tests. About 26% of the mTBI patients showed declining performance on the Mini-Mental State Examination (MMSE), reflecting overall cognitive deficits [30]. Furthermore, episodic memory impairment was more pronounced than executive function difficulties [30]. Our study corroborated significant differences in overall cognitive function tests among mTBI patients, yet the statistical significance was less pronounced in the Y-maze and MWM tests compared to the NOR test. Nonetheless, patients treated with α-pinene consistently exhibited improved cognitive performance, suggesting its potential utility in reducing cognitive impairments associated with mTBI.

One of the pivotal issues related to the clinical α-pinene use is the optimal route of administration. While we opted for the IV route, most studies have analyzed the therapeutic effects of α-pinene via intraperitoneal (IP) injection [4,27]. An IP injection involves delivering α-pinene into the peritoneal cavity, and subsequently into the systemic blood circulation via peritoneal and interstitial lymphatics [31]. Although this method is easier and faster than the IV method, it may not be suitable for use in humans. Additionally, the distribution of treatment molecules varies according to the administration route in in vivo conditions [32]. Dou et al. reported about a 30-fold higher absorbed radiation dose due to slow entry into the circulation, resulting in inadequate targeted tumor accumulation [33]. Although the mechanisms and environments of TBI and tumor diseases differ, such findings suggest that it may be more advantageous to test the α-pinene effect via the IV route than the IP route, considering clinical translation in the real world.

There are some limitations in this study. Firstly, we administered α-pinene immediately after brain injury and maintained the treatment for 3 days. Importantly, no mortality or overt signs of systemic toxicity were observed in any treatment group throughout the experimental period. Although medicinal plant-derived compounds are increasingly considered as alternatives to conventional painkillers, concerns remain regarding their potential hepatotoxicity during long-term use. Previous studies have reported both the safety and therapeutic efficacy of such compounds [34,35], but yet the risk of hepatotoxicity continues to be a challenge for clinical application. Consequently, we treated mTBI mice with α-pinene only for a short period post-injury without observing any changes in liver enzymes (Supplemental Figure S1). Further research is required on the duration of α-pinene treatment to enhance therapeutic efficacy. Secondly, no direct comparison between α-pinene and conventional drugs was conducted. The current conventional treatment for mTBI involves nonsteroidal anti-inflammatory drugs (NSAIDs). Although an analysis of 16 relevant cohort studies [36] suggested that NSAIDs might aid cognitive impairment in patients with AD, there is no clear evidence of a therapeutic effect of anti-inflammatory drugs on outcomes in those with TBI [37]. In fact, chronic use of NSAIDs exacerbated cognitive impairment in a rodent model of TBI [38]. Despite these controversial results, NSAIDs remain commonly prescribed to patients with mTBI. Thus, comparative analyses between α-pinene and NSAIDs are warranted. The present findings suggest that α-pinene may represent a promising therapeutic candidate for the management of mTBI-related neurological dysfunction.

### Future Research

Future studies are required to further investigate the optimal therapeutic window, long-term safety, and dose–response relationship of α-pinene in TBI models. In addition, comparative studies evaluating α-pinene against currently used medications, including nonsteroidal anti-inflammatory drugs (NSAIDs) and beta blockers, may help clarify its translational potential in clinical practice. Importantly, the current study may contribute to addressing several unmet clinical needs in patients with mTBI, particularly those related to persistent cognitive dysfunction and insufficient therapeutic options, as highlighted in the Dasic matrix for neurotrauma services in a major trauma center [39]. These findings support the potential clinical relevance of α-pinene as a novel therapeutic strategy for mTBI.

## 5. Conclusions

Our study suggested that α-pinene has the potential to serve as an effective alternative to manage symptoms not fully controlled by conventional drugs in mTBI. Administering α-pinene immediately post-injury may confer neuroprotection and enhance cognitive performance through the decrease in NOX4 activity.

## Data Availability

The original contributions presented in this study are included in the article. Further inquiries can be directed to the corresponding author(s).

## Supplementary Materials

The following supporting information can be downloaded at: https://www.mdpi.com/article/10.3390/life16071110/s1, Supplemental Table S1: Primer sequences for quantitative real-time polymerase chain reactions; Supplemental Figure S1: (A–C) Alterations in body weight, serum AST, and serum ALT following treatment with α-pinene; Supplemental Figure S2: (A–C) Original uncropped Western blot images used for Figure 2B; Supplemental Figure S3: (A–C) Original uncropped Western blot images used for Figure 3F; Supplemental Figure S4: (A,B) Original uncropped Western blot images used for Figure 4F.
