# Supplementary figures and images for: Therapeutic Effect of Alpha-Pinene on In Vitro and In Vivo Models of Mild Traumatic Brain Injury
Source: Life (Basel). 2026 Jul 2;16(7):1110. doi: 10.3390/life16071110 (PMC13412431; doi:10.3390/life16071110)

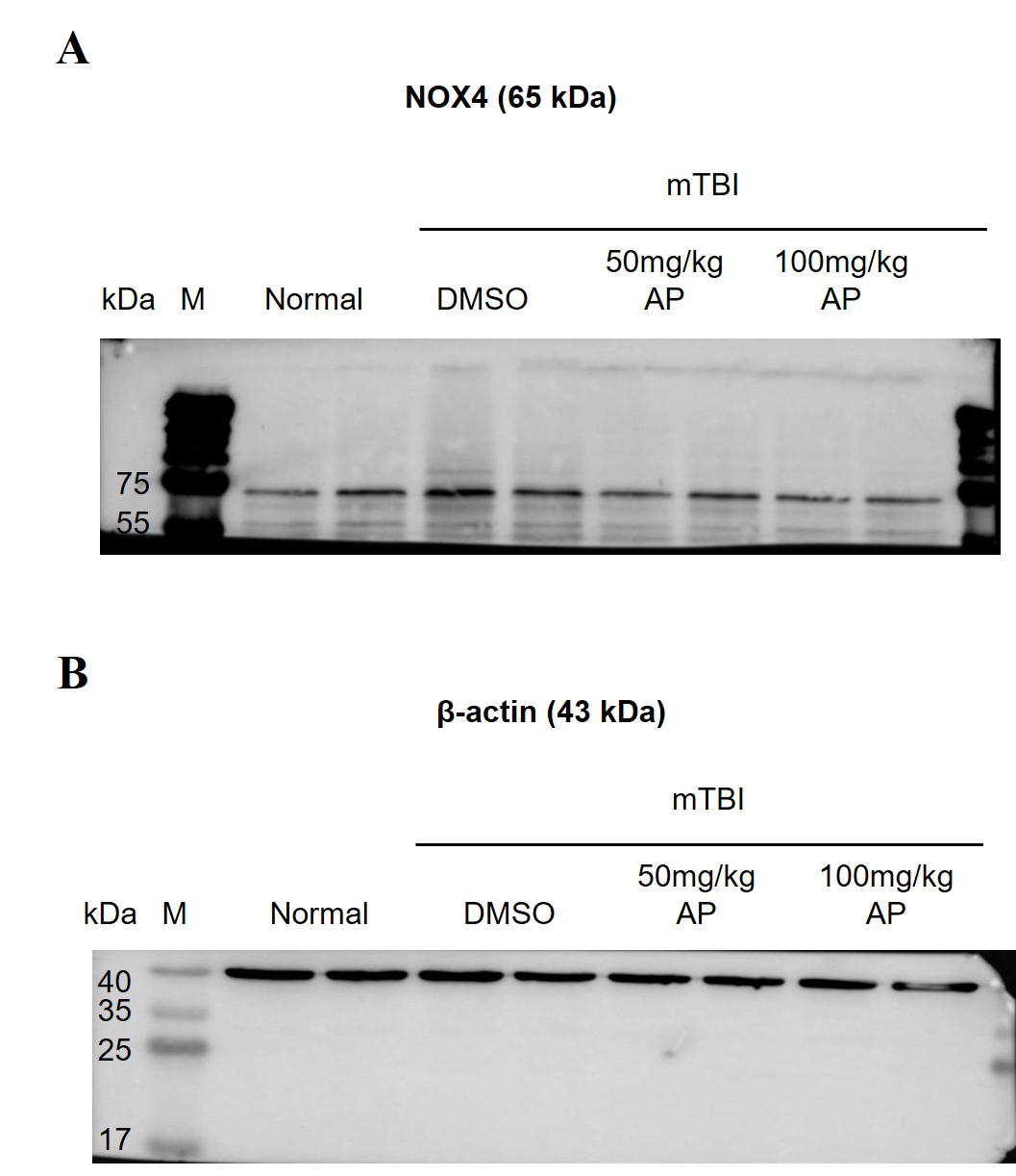

Supplement: Supplementary file 1 [file life-16-01110-s001.zip › Supplementary Figure S4.jpg]

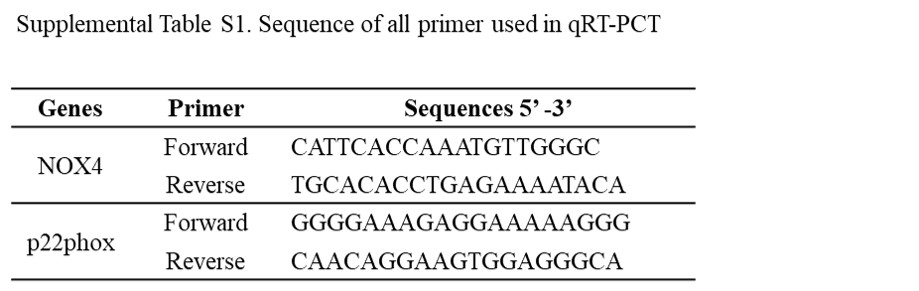

Supplement: Supplementary file 1 [file life-16-01110-s001.zip › Supplementary Table S1.jpg]

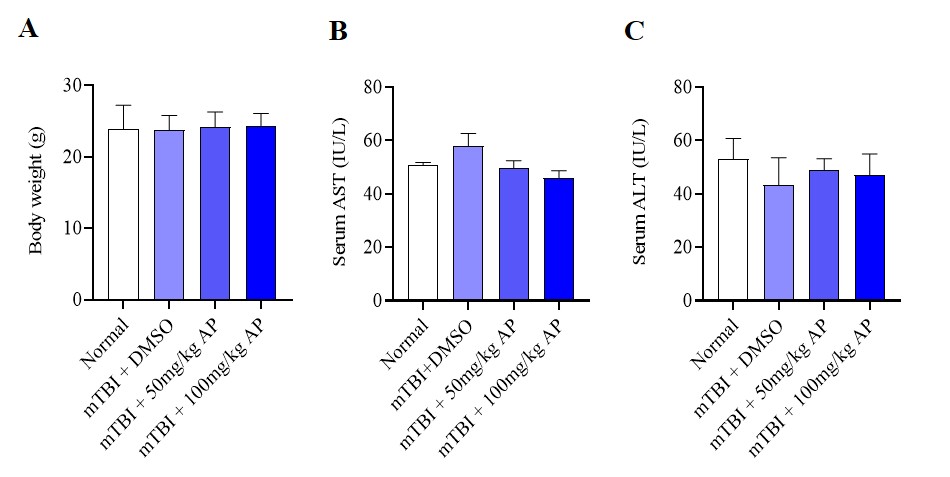

Supplement: Supplementary file 1 [file life-16-01110-s001.zip › Supplementary Figure S1.jpg]

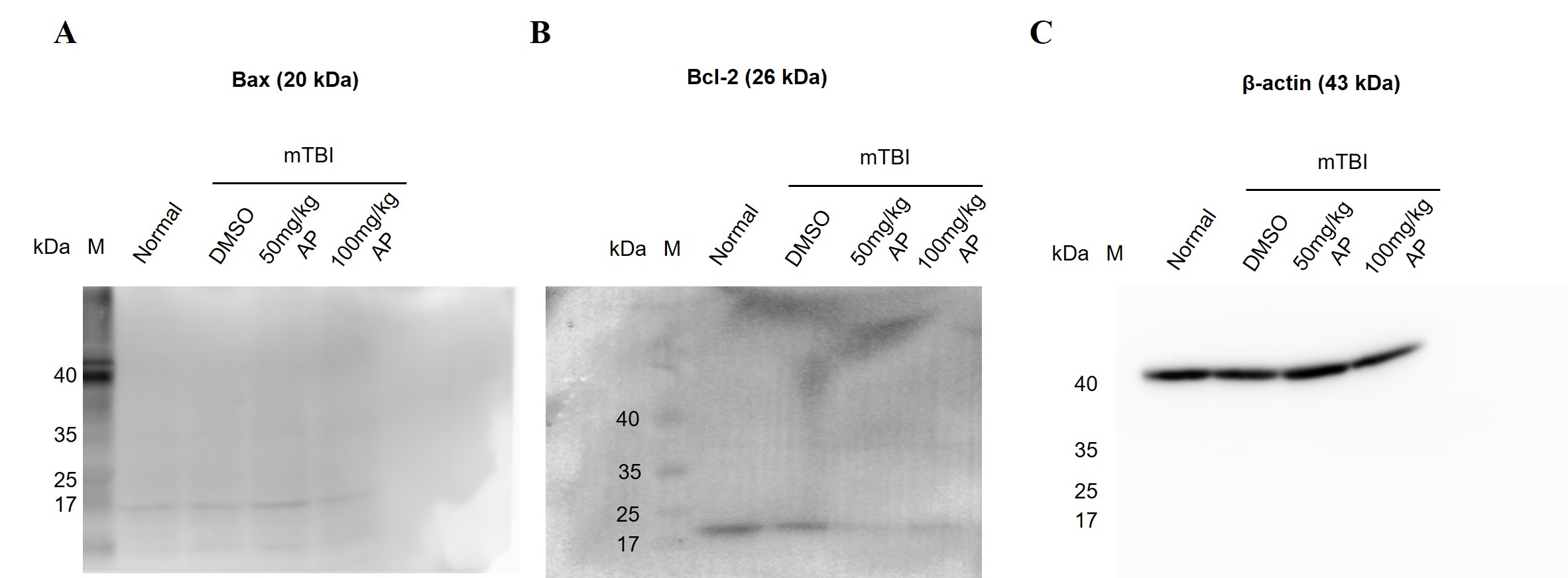

Supplement: Supplementary file 1 [file life-16-01110-s001.zip › Supplementary Figure S2.jpg]

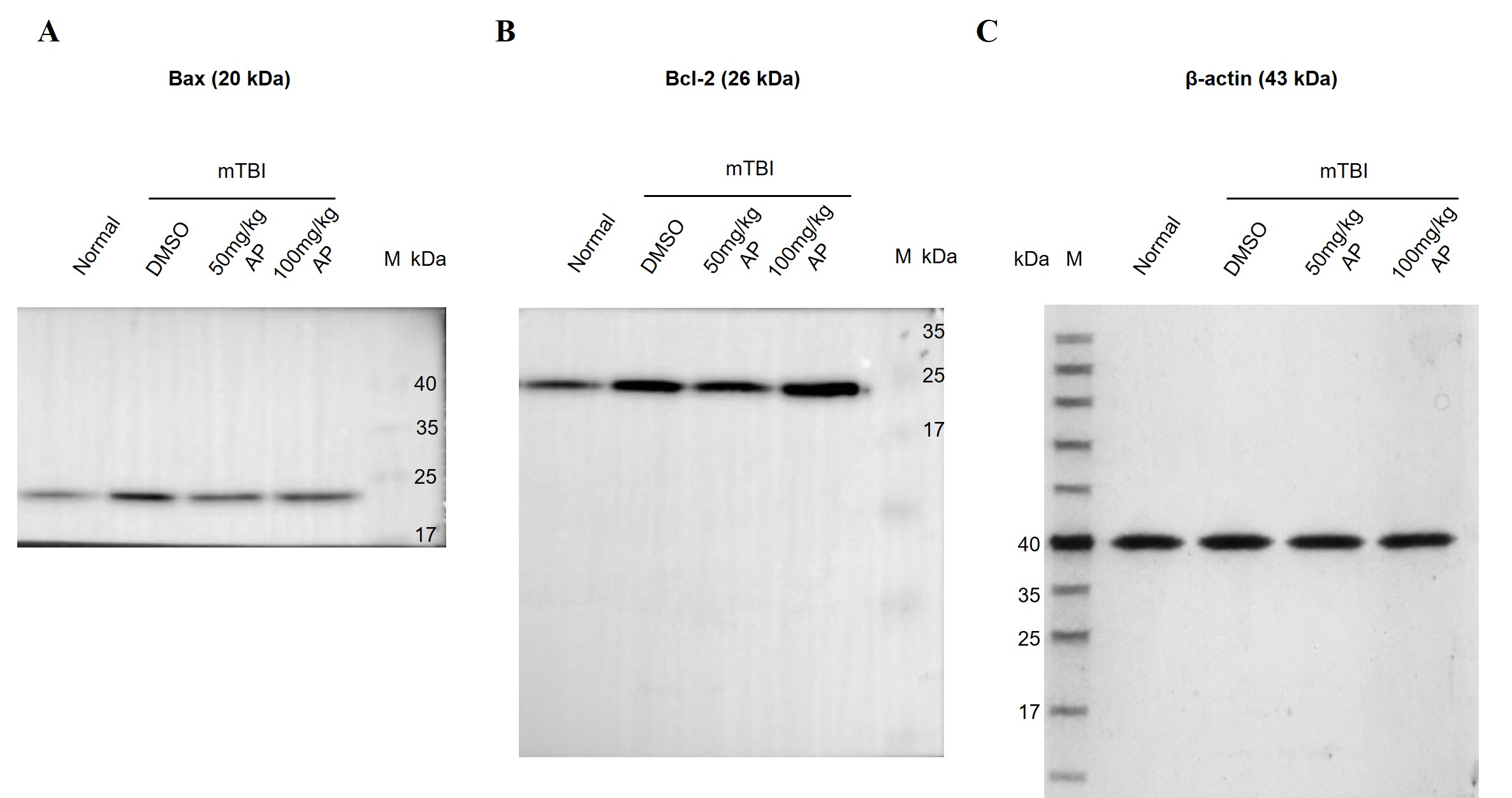

Supplement: Supplementary file 1 [file life-16-01110-s001.zip › Supplementary Figure S3.jpg]
